# Supplementary material for: The effects of an invasive seaweed on native communities vary along a gradient of land-based human impacts
Source: PeerJ. 2016 Mar 21;4:e1795. doi: 10.7717/peerj.1795 (PMC4806595; doi:10.7717/peerj.1795)
Supplement: Supplemental Information 3 — Linear-mixed models assessing the effects of C. cylindracea, land-based- and sea-based cumulative human impacts on the species richness of developing assemblages after the exclusion of a potential outlier. Coefficients, standard errors (SE) and p-values are reported for fixed effects, while variance (δ2) and standard deviation (SD) are reported for random effects. [file peerj-04-1795-s003.docx]

Table S1. Linear-mixed models assessing the effects of *C. cylindracea*, land-based- and sea-based cumulative human impacts on the species richness of developing assemblages after the exclusion of a potential outlier. Coefficients, standard errors (*SE*) and *p*-values are reported for fixed effects, while variance (*δ*^2^) and standard deviation (*SD*) are reported for random effects.

Effect Estimate *SE*/*SD* *P*

*Fixed effects*

Intercept 28.058 6.678 **0.002**

*- C. cylindracea = - C.* -3.423 4.213 0.419

Land-based score = L -2.187 0.511 **0.002**

Sea-based score = S -2.723 1.514 0.103

- *C.* x L -0.781 0.336 **0.023**

- *C.* x S 1.373 0.958 0.156

*Random effects δ*^2^ *SD*

Site 2.585 1.608

Residual 3.211 1.792
