# Supplementary material for: The effects of an invasive seaweed on native communities vary along a gradient of land-based human impacts
Source: PeerJ. 2016 Mar 21;4:e1795. doi: 10.7717/peerj.1795 (PMC4806595; doi:10.7717/peerj.1795)
Supplement: Supplemental Information 4 — Table S2–ANOVA on the effects of Site (3 levels, random), Assemblage (control versus cleared; fixed) and C. cylindracea (present versus removed; fixed) on the percentage cover of canopy-forming species. Pooling procedures were used, according to Winer et al. (1991). [file peerj-04-1795-s004.docx]

Table S2. ANOVA on the effects of Site (3 levels, random), Assemblage (control versus cleared; fixed) and *C. cylindracea* (present versus removed; fixed) on the percentage cover of canopy-forming species. Pooling procedures were used, according to Winer et al. (1991)

Source of variation df MS *F* *P*

Site = S 2 29.218 27.86 0.000

Assemblage = A 1 2.237 1.10 0.405

*C. cylindracea* = *C* 1 9.264 8.83 **0.005**

S x A 2 2.035 1.94 0.155

S x *C*^a^ 2 eliminated

A x *C* 2 0.339 0.110 0.768

S x A x *C* 2 2.981 2.84 0.068

Residual 48 1.049

Transformation *C*-test

Ln(x+1) 0.213; p > 0.05

^a^Tested against the Residual

References

Winer B.J., Brown D.R., Michels K.M. 1991. Statistical principles in experimental design. McGraw-Hill, New York
